# 416. Diagnostic Yield of Serial COVID-19 Testing in Hospitalized Patients

**DOI:** 10.1093/ofid/ofab466.616

**Published:** 2021-12-04

**Authors:** Jeremy Li, Charles Frenette, Vivian Loo

**Affiliations:** 1 McGill University, Montreal, Quebec, Canada; 2 McGill University Health Centre, Montreal, Quebec, Canada

## Abstract

**Background:**

Accurate and rapid diagnosis of SARS-CoV-2 infection is essential to prevent nosocomial transmission. Patients with negative COVID-19 tests at admission may still be in the incubation phase during hospitalisation. False negative results can occur when patients are tested too early. The incidence of COVID-19 infections in Montréal, Canada started to increase in December 2020. Because of this rise, on January 4^th^, 2021, the Infection Control Service of the McGill University Health Centre (MUHC) recommended serial COVID-19 testing for all admitted patients on days 5 and 10 after admission. The aim of this study is to examine the diagnostic yield of serial COVID-19 testing.

**Methods:**

We retrospectively analyzed SARS-CoV-2 test results for patients admitted to the MUHC between January 4, 2021, and April 30, 2021. Nasopharyngeal swabs were collected from patients for SARS-CoV-2 PCR testing. Multiple testing platforms were used (Roche Cobas 6800, Thermo Scientific^TM^ King Fisher and Cepheid GeneXpert) because of the high volume of samples. Tests were classified as admission, day 5, and day 10 tests if they were done on days 0 to 2, 3 to 7, and 8 to 12 respectively. Patients positive for SARS-CoV-2 on admission were excluded from the analyses. The diagnostic yield of serial testing for patients admitted during each month was calculated by dividing the number of patients testing positive on day 5 or day 10 by the total number of patients who underwent serial testing during that month.

**Results:**

There were 2945 admissions of 5 days or more and 1777 admissions of 10 days or more. Of these, 1509 patients and 841 patients respectively were serially tested for SARS-CoV-2 as recommended for a compliance rate of 51% at day 5 and 47% at day 10. Ten (0.7%) and 12 (1.4%) patients tested positive on days 5 and 10 respectively. The diagnostic yield of serial testing was highest for patients admitted in January 2021 at 2.2%, when the average daily incidence of COVID-19 was highest in Montréal (see Figure).

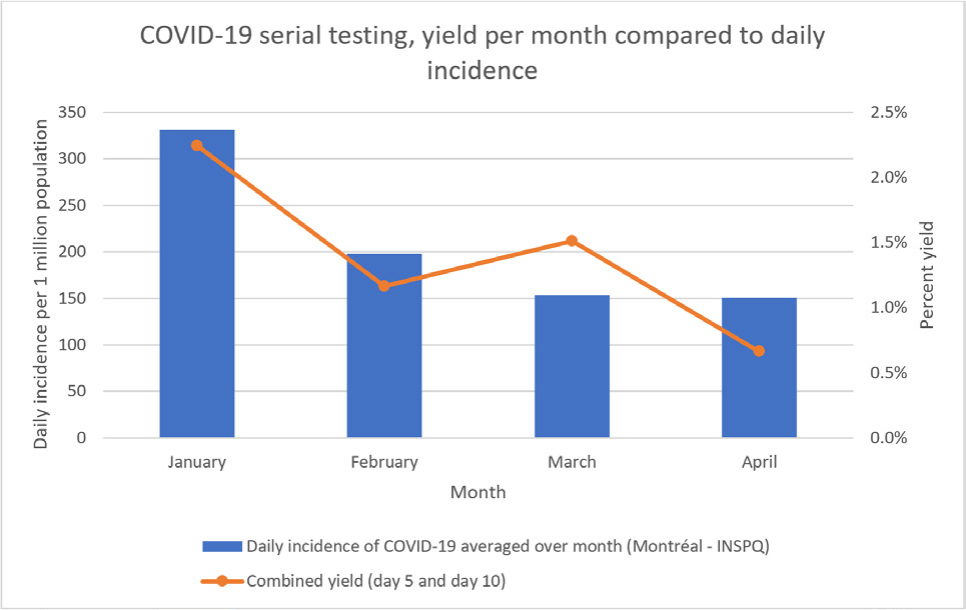

The diagnostic yield of serial testing for each month, compared to the average daily COVID-19 incidence rate in Montréal, Québec, Canada.

**Conclusion:**

The diagnostic yield of serial SARS-CoV-2 testing in hospitalized patients is low when the overall community incidence is low. However, diagnostic yield of serial testing increases when community incidence of COVID-19 is higher and should be considered in this situation.

**Disclosures:**

**All Authors**: No reported disclosures

